# The effect of recall, reproduction, and restudy on word learning: a pre-registered study

**DOI:** 10.1186/s40359-017-0198-8

**Published:** 2017-08-04

**Authors:** Saloni Krishnan, Kate E. Watkins, Dorothy V.M. Bishop

**Affiliations:** Department of Experimental Psychology, University of Oxford, South Parks Road, Oxford, OX1 3UD UK

**Keywords:** Testing effect, Production effect, Retrieval, Nonword learning

## Abstract

**Background:**

Certain manipulations, such as testing oneself on newly learned word associations (recall), or the act of repeating a word during training (reproduction), can lead to better learning and retention relative to simply providing more exposure to the word (restudy). Such benefit has been observed for written words. Here, we test how these training manipulations affect learning of words presented aurally, when participants are required to produce these novel phonological forms in a recall task.

**Methods:**

Participants (36 English-speaking adults) learned 27 pseudowords, which were paired with 27 unfamiliar pictures. They were given cued *recall* practice for 9 of the words, *reproduction* practice for another set of 9 words, and the remaining 9 words were *restudied*. Participants were tested on their recognition (3-alternative forced choice) and recall (saying the pseudoword in response to a picture) of these items immediately after training, and a week after training. Our hypotheses were that reproduction and restudy practice would lead to better learning immediately after training, but that cued recall practice would lead to better retention in the long term.

**Results:**

In all three conditions, recognition performance was extremely high immediately after training, and a week following training, indicating that participants had acquired associations between the novel pictures and novel words. In addition, recognition and cued recall performance was better immediately after training relative to a week later, confirming that participants forgot some words over time. However, results in the cued recall task did not support our hypotheses. Immediately after training, participants showed an advantage for cued *Recall* over the *Restudy* condition, but not over the *Reproduce* condition. Furthermore, there was no boost for the cued *Recall* condition over time relative to the other two conditions. Results from a Bayesian analysis also supported this null finding. Nonetheless, we found a clear effect of word length, with shorter words being better learned than longer words, indicating that our method was sufficiently sensitive to detect an impact of condition on learning.

**Conclusions:**

Our primary hypothesis about training conditions conferring specific advantages for production of novel words presented aurally, especially over long intervals, was not supported by this data. Although there may be practical reasons for preferring a particular method for training expressive vocabulary, no difference in effectiveness was detected when presenting words aurally: reproducing, recalling or restudying a word led to the same production accuracy.

## Background

People constantly encounter and learn new words, for example, the smattering of foreign language words we pick up while travelling to other countries, the names of companies that perform new functions in our everyday lives (e.g. “Google”), or words we encounter in games (for instance, names of Pokémon in popular game Pokémon Go). By adding these words to our vocabulary, we can talk about new ideas and concepts. Learning these new words is not trivial at the cognitive level, and comprises multiple components, such as matching a word to a referent [1], developing a deeper understanding of the referent, as well as learning a novel sequence of sounds [2, 3]. The latter form of learning is critical for production. In order to produce a word, it is important not just to recognise a sequence of sounds, but achieve near perfect recall^1^ of it. In fact, children with developmental language disorders particularly struggle with the sequential and phonological aspect of learning in verbal-visual association tasks [4, 5]. Therefore, there is a pressing need to examine if and how the ability for recall of new phonological forms can be optimised, and how this affects novel word learning.

Although many words are implicitly extracted and learnt in contextually rich environments through repeated exposure, there are more explicit ways that words might be taught. In the literature on learning and memory, certain manipulations are known to lead to better retention for words encountered during reading. For example, Karpicke and Roediger [6] demonstrated that when English speakers had to learn 40 pairs of English-Swahili words, their learning was enhanced for items they had to recall during a test relative to items they had merely restudied. After 1 week, participants could recall 80% of word pairs they were repeatedly tested on, but only 33–36% of word pairs they had repeatedly restudied. The conclusion was that the act of recall in testing scenarios leads to better learning, as assessed by recall tasks [7–9]. The ‘testing effect’ refers to the notion that recalling information, or engaging in a test, instead of restudying the material, serves as a potent learning event that is of critical importance for learning [10]. The testing effect is a well-studied phenomenon that is considered quite reliable [11, 12]. It has been demonstrated using a range of material to be learned, including verbal and non-verbal material [13]. Recall has been trained using both spoken and typed responses; the nature of retrieval does not appear to reduce the magnitude of the testing effect [14, 15]. Interestingly, however, the benefit of recall during the learning phase is sometimes noted at a later delay rather than immediate retention [16–18]. Multiple theories have been put forward to explain the testing effect. For example, the transfer-appropriate processing theory suggests that testing effects stem from the similarity of the learning processing during testing and the final assessment [19]. In a general sense, theories of this kind maintain that tests allow an opportunity to practice encoding and retrieval in a manner that is the optimal for the final test [20]. As such, they are broadly compatible with the retrieval effort or bifurcation hypotheses discussed below. However, the more specific formulation is that testing effects arise when there is a high degree of similarity between learning and assessment. However, a recent meta-analysis [11] demonstrated that the extent of the initial-final test match was not associated with the magnitude of the testing effect. Rather, more ‘difficult’ initial tests, such as free recall, tended to boost performance on all other tests [21]. This links to theories of retrieval effort, which maintain that the cognitive effort expended during the initial test strengthens memory for the item [22]. Other theoretical accounts of the testing effect propose it is influenced by semantic elaboration during retrieval (elaborative retrieval hypothesis [23], or mediator effectiveness hypothesis [24]). A more recent theory is the bifurcation hypothesis, which suggests that all items start with the same memory strength, but that successful retrieval greatly increases the memory strength of tested items, leading to two distinct distributions of tested and non-tested items [25]. In a recent meta-analysis, Rowland [11] demonstrated that the bifurcation and retrieval effort hypotheses best fit experimental data, with elaborative processing suggested as a mechanism that fits with the data, but one which cannot stand alone.

Another manipulation thought to influence word learning is production. There is some existing evidence that imitation or reproduction of words in a foreign language improves expressive learning of the word relative to imagery [26], and also relative to repeated restudy [27]. However, this effect has been more systematically studied in relation to reading known words. Here, the “production effect” refers to the phenomenon that producing a word aloud during study, relative to simply reading it silently, improves memory for the item [28, 29]. Production has recently been shown to boost recall of the produced item and also the connections between a produced item and a related item in an association task [30]. Interestingly, this effect is consistently observed when manipulated within subjects, but not between subjects. It is not confined to overt production; as silent mouthing of a word also confers the same advantage. The production effect is also observed for reading pseudowords, indicating that an item does not have to have a pre-existing lexical entry to get this benefit. In a recent study, adults were taught pseudowords, some of which they repeated during learning whereas some were only heard. Later testing revealed that participants were quicker at recognising the pseudowords that they had produced during training [31]. The production effect is thought to result from distinctiveness conferred on the word by virtue of pronouncing it [28]. An alternative account is that these effects result from motor prediction mechanisms that support learning [32]. Understanding the effects of these manipulations on learning novel expressive vocabulary is not just of theoretical interest; it also has relevance in clinical or educational settings.

These two explicit manipulations, testing and production, share some similarities. First, both are typically contrasted against a ‘*Restudy’* condition, which involves gaining more exposure to the item by repeatedly studying it. This is typically what students do in classroom settings. In contrast to the “restudy” condition, both testing (operationalized by getting participants to engage in cued *recall* of the item) and production (operationalized by making participants *reproduce* the item) involve active and effortful manipulation of the information to be learnt. Second, recall and reproduction are also relatively naturalistic methods of training and are employed in classroom learning; for instance, testing via flashcards or repetition of what the instructor says. They are also typical forms of practice in modern language learning apps, such as DuoLingo or Rosetta. Third, *Recall* and *Reproduction* both involve overt generation of an item, in contrast to the *Restudy* condition, so that participants have a chance to encode the motor/ kinaesthetic properties of the word.

There are, however, also differences between the testing and reproduction conditions. First, although both these conditions involve generating a spoken word, they tap retrieval in different ways. While testing (*Recall*) involves retrieval without access to the item, thereby creating an elaborative memory trace, production (*Reproduce*) taps mainly short-term memory processes [33]. This could explain why testing is associated with better recognition and recall *over time,* with some studies even noting that restudy leads to better performance immediately after learning [6, 16]. This profile has not generally been noted for production, which is mainly associated with improved performance at *immediate* recognition and recall. An exception is a study by Ozbuko and colleagues [34] who found that a production effect was observed on a yes/no recognition test one-week after a delay, but it is unclear if these effects would be seen in a more difficult cued-recall test, which involves remembering the exact sequence of sounds in the target.

Testing is also thought to require more cognitive effort than reproduction [35]. Furthermore, test instructions may lead to participants using different retrieval modes. In a study contrasting intentional word retrieval to incidental word retrieval (as tapped in a condition that involved completing fragments of words via guessing any answer or explicitly retrieving the cue associated with the target), Karpicke and Zaromb [36] found that intentional retrieval led to greater retention relative to generation. The authors argued that incidental retrieval may involve a more implicit learning strategy, whereas intentional retrieval forced participants to rely on episodic retrieval of events.

Finally, the two training manipulations (*Recall* and *Reproduction*) are also associated with slightly different neurobiological substrates. Overtly recalling a correct word during testing is associated with activation in the right hippocampus [37]. Wing, Marsh and Cabeza [38] also looked at neural processing of words that were subsequently remembered or forgotten when trained via recall or restudying. They observed greater activity in the parahippocampal gyrus for training via test and recall relative to restudying, and differences in activity in the hippocampus bilaterally when they examined the interaction between training condition and subsequent memory. More specifically, they found that both the left and right hippocampus showed more activity during successful encoding for the test trials relative to the restudy trials. The role of medial temporal lobe structures during recall is also thought to involve updating representations with relevant new information about cue-target associations. This process supports more efficient search for the target when presented with a cue. Medial temporal lobe regions also interact with cortical regions to create an enduring representation of this association. One form of information about a target word is a novel articulatory or phonological sequence. A few neuroimaging studies have suggested that learning articulatory/ phonological form is typically supported by corticostriatal regions. For example, when repeating novel words relative to known words, there is a decline in activity in striatal regions such as the left and right caudate nucleus [39]. A similar decline of activity has been observed in the putamen during covert vocal learning of nonwords [40]. Repeated word production would allow learners to create a rich sensorimotor representation of the item, which would be unavailable for items that were not produced overtly or covertly. Recently, we suggested that altering the extent to which training conditions depend on different neurobiological systems could lead to differences in learning performance [41].

The current study was designed to test this idea, by evaluating the impact of *Recall* (the testing effect) and *Reproduction* (the production effect) on novel word learning, relative to *Restudy*. As we presented words aurally, we operationalised conditions differently to studies that use visual presentation. In our variant of the *Restudy* condition, participants heard a pseudoword on each trial in conjunction with a visual referent. Immediately after the auditory exposure, they were prompted to say “okay” as the response. This manipulation was introduced to limit covert practice of the pseudoword, as covert retrieval may lead to the same outcomes as overt retrieval [14, 15]. Furthermore, this manipulation allowed us to match conditions so that all involved overt speech production along with monitoring of feedback of self-produced speech. In the *Reproduce* condition, participants heard a pseudoword on each trial in conjunction with a visual referent. Immediately after the auditory exposure, they were prompted to repeat the word out loud. Finally, in the cued *Recall* condition, participants were only presented with the visual referent and were prompted to retrieve the pseudoword from memory. We assessed retention of the pseudowords immediately after training and a week later, using both a recognition and a cued-recall test. Our procedure differs in some ways from previous studies that have studied testing and production effects. As we are interested in learning of the phonological form of novel spoken words, participants never encountered the written form of the words they were meant to learn. This is because orthographic representations can allow access to a phonological representation, and particularly in good readers, the presence of orthography improves word learning [42, 43]. Instead, participants had to create a stable phonological representation from auditory exposure alone. We used pseudowords of varying lengths (2-, 3-, and 4-syllables) to guard against floor or ceiling effects in production. Varying word complexity in this manner also allowed us to assess whether we obtained the classic word length effect for pseudowords in this task [44], which would provide a positive control for our paradigm, by demonstrating that the phonological forms of these words are learnt in expected ways. Finally, when participants were being trained (and at the final tests immediately after training and a week following training), they generated spoken responses. Based on the literature highlighted above, we would expect that at the immediate time-point, participants would have more accurate recognition and cued-recall for words they for which they had more auditory exposures, that is, the *Restudy* and the *Reproduce* conditions. We would also expect that accuracy for words in the cued *Recall* condition would be enhanced relative to *Restudy* and *Reproduce* 1 week after training. Although we made no a priori predictions about this, it would be reasonable to expect to see that performance on words learned in the *Reproduce* condition would be higher than for those learned in *Restudy* at the delayed time-point, because of the greater amount of processing expended in the initial learning of these words [45]. That is, participants make more decisions about a given item in this condition. This prediction would also be unsurprising under accounts of the production effect, which argue that the words are conferred greater distinctiveness when repeated [30].

We pre-registered the following predictions for this task on the Open Science Framework (https://osf.io/6n9df/register/565fb3678c5e4a66b5582f67). Note that we have renamed conditions to make this manuscript easier to follow, and to clearly distinguish between processes of retrieval and cued recall.^2^

1. In the testing session that takes place on the day of training, we expect that the training conditions that involve *Restudy* or spoken *Reproduction* (rather than cued *Recall*) will lead to greater accuracy in a 3-alternative forced choice (3AFC) task assessing recognition and enhanced performance in a production task assessing cued recall, due to the greater number of listening exposures in these conditions.
2. In the testing session that occurs a week after training, the training condition that involves cued *Recall* (relative to *Reproduction* or *Restudy*) will be associated with greater accuracy in a 3AFC task assessing recognition and enhanced learning in a production task assessing cued recall.
3. In both sessions, recognition and cued recall accuracy will be greater for shorter pseudowords relative to longer pseudowords.

## Methods

### Ethics

The University of Oxford Medical Sciences Division Research Ethics Committee approved this study (approval reference: R37093/RE001). All participants gave written informed consent prior to participation.

### Data and material release

The training program for this study and raw data, along with details of the analyses run using JASP Stats are available on https://osf.io/cjx4e/

### Participants

To calculate the appropriate sample size for this repeated measures analysis, we used procedures described by Guo and colleagues [46], instantiating them in the GLIMMPSE calculator available at http://glimmpse.samplesizeshop.org/. This requires users to enter a sample set of means. Extrapolating from previous studies [6, 30], we estimated that at Week 0, the participants would correctly recall 60% words in the cued *Recall* and *Restudy* conditions, and that this would be increased in the *Reproduce* condition to 80% words. At Week 1 (i.e. a week after training), we estimated that 80% of words learned in the cued *Recall* condition would be produced accurately, whereas in both the *Reproduce* and *Restudy* conditions, 60% of the learned words would be remembered. Within-participant correlations across condition were estimated at 0.5 for condition and time, and response variability was assumed to be 15%. To obtain main effects of condition, time and the condition x time interaction at 0.8 power, the highest estimate (taking into account double the variability, 30%) was 34 participants. This number was also sufficient to address our question about word length. We therefore decided on a target sample size of 36 participants, which allowed us to fully counterbalance our conditions.

We recruited 36 healthy volunteers between the ages of 18–40 years who spoke English as a first language, using the departmental participant pool at the University of Oxford, and via advertisements displayed on noticeboards around the department. Data from one participant was removed at stage 1 (as his language questionnaire indicated that he did not speak English as a first language); we replaced this participant. There was no further attrition; all participants complied with instructions and completed all the tests. Therefore, thirty-six participants completed this experiment and received a small payment for their participation (for further demographic details, see Table 1).

**Table 1:** Participant details. For age, WASI scores and CVLT-II free recall scores, the mean is provided and standard deviation is indicated in brackets

Measure	
N	36 (9 males)
Number of languages spoken	Median: 2, Range: 1–5
Age (years)	24.74 (4.9)
WASI Matrix Reasoning (T-score equivalent of raw score)	58.44 (5.1)
CVLT-II free recall total score (Raw score; maximum = 100)	70.06 (7.0)

In exploratory analyses (listed on our OSF pre-registration), we also wanted to assess whether variation in participants’ age, IQ, the number of languages they spoke, or their verbal memory would correlate with their word learning ability. Consequently, participants also completed the Language Experience and Proficiency Questionnaire (LEAP-Q [47]) to assess their language background, the California Verbal Learning Test (CVLT-2; [48]) to assess verbal memory, and the Matrix Reasoning subtest of the Wechsler Abbreviated Scale of Intelligence (WASI; [49]) to assess nonverbal reasoning.

### Word learning task

This task was designed to assess the influence of training condition (*Recall/Reproduction/Restudy*) and word length (2-, 3-, and 4-syllables) on cued recall and recognition accuracy assessed at two time-points (Week 0, immediately after training; and Week 1, a week after training).

### Stimuli

Visual stimuli were chosen from a commercially available image database (shutterstock.com). We picked 15 pictures of sea creatures, 15 pictures that showed undersea plants, and 15 pictures of seashells. The pictures were chosen to be easily distinguishable and to belong to separate categories. A further consideration was that they should not be associated with familiar verbal labels (for example, goldfish). After pilot testing, 9 pictures in each category were retained.

Three pseudoword lists, comprising 9 words each, were used in this study. Each list consisted of 3 two-syllable words, 3 three-syllable words, and 3 four-syllable words. The first list consisted of a subset of words drawn from the Children’s Test of Nonword repetition (CNRep). The other two lists were drawn from two pseudoword lists constructed for a previous study with children and matched with respect to number of syllables, stress pattern, and consonant clusters (Hobson, unpublished work). Pilot testing established that the lists were matched in difficulty.

### Randomisation

The list order for the pseudowords was fixed, such that List 1 always occurred first, List 2 s and List 3 last. Although list order was fixed, within each list, the order of the words was random, so the words did not occur in the same order each time. The pictorial stimuli paired with each pseudoword list changed across participants. For instance, Pseudoword List 1 could be paired with Picture List 1 (creatures), 2 (plants), or 3 (shells). There were 6 possible permutations of these pairings. This meant that participants learnt different pairings of pseudowords-pictures, but the pairings were consistent within each participant. There were also three training conditions – ‘*Recall’*, ‘*Reproduce’*, and ‘*Restudy’*. Six training orders were constructed which comprised all permutations of these conditions. Taken together, 36 permutations of training method and word-picture pairing order were created. Each participant was randomly assigned to one of these permutations.

### Procedure for training phase (fig. 1)

**Fig. 1:**
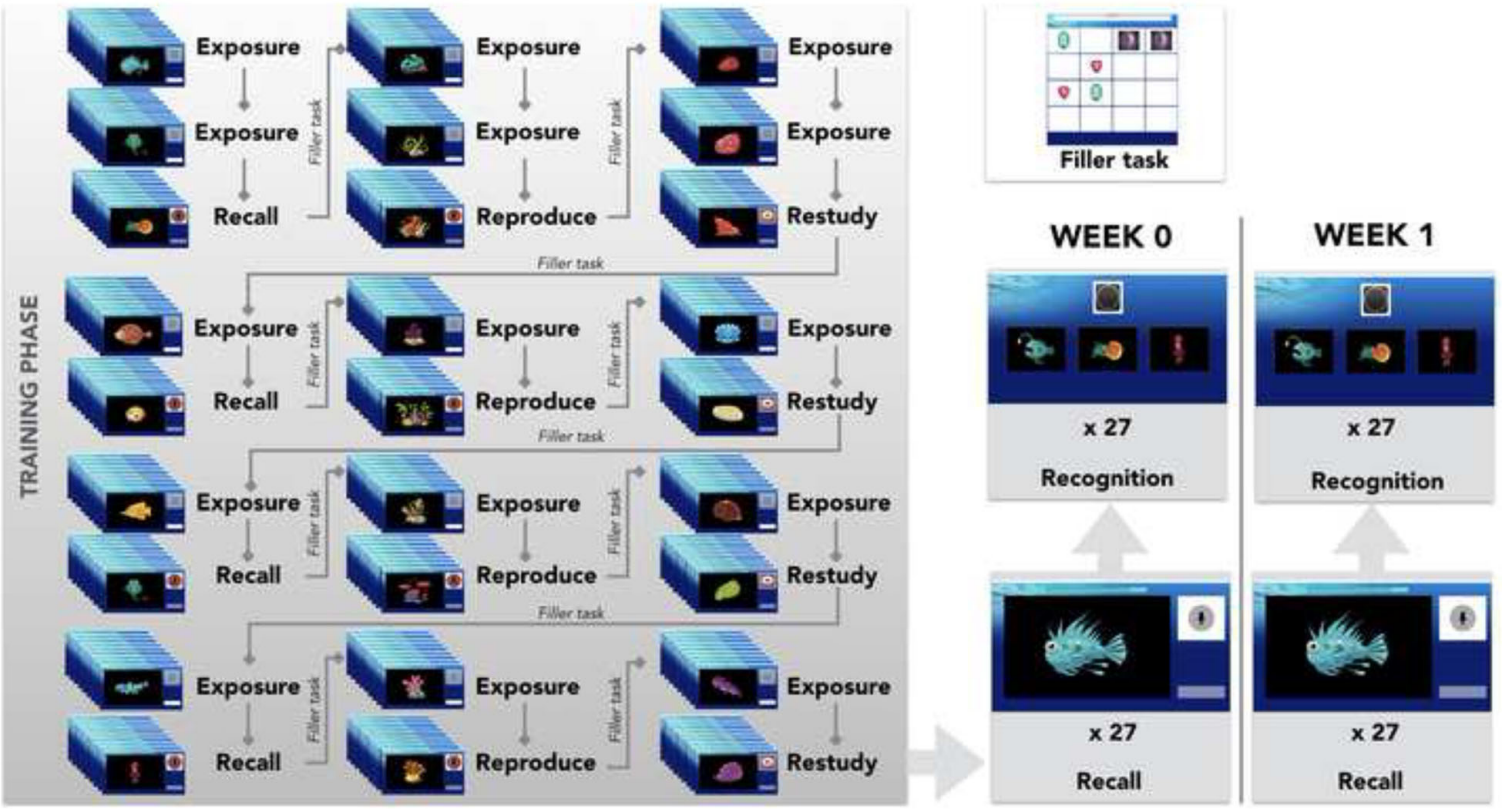
Task Schematic. Task structure for a single session is depicted here. In this run, creatures are the first category to appear, followed by the plants, and then the seashells. Creatures are associated with the retrieval condition, plants with the reproduction condition, and shells with the restudy condition. The arrows illustrate how participants cycle through the exposure and training phase for each condition (Recall, Reproduce, and Restudy). Within each block, the order of trials is randomly determined. Some blocks are followed by a filler task, which involves finding pairs of matching pictures (as illustrated in the top right corner). At the end of the training phase, the participants’ cued recall and recognition for all 27 novel word-picture associations are tested. A week later, participants only complete the cued recall and recognition task; they are not exposed to the training phase

Participants were instructed to learn the names of 9 creatures, 9 plants, and 9 shells during the training phase. They were told that they had not heard these names before, and they needed to follow the on-screen instructions in order to learn them. A schematic of the training procedure participants completed is shown in Fig. 1.

The order of the three training conditions *Recall*, *Reproduce*, and *Restudy* was counterbalanced across participants. Before the first training condition, participants were exposed to the stimuli to be learned in that condition. At the start of the exposure block, participants were told to simply listen carefully and try to learn the name of the picture. For each exposure trial, they were shown a picture on screen as they heard the pseudoword associated with the picture. Once the trial was done, they simply had to click a button to move to the next trial. These were provided so participants would have some familiarity with the words. At the start of each block of the training trials, participants were given instructions specific to the condition. For the *Restudy* condition, they were told to listen carefully to the word. They were explicitly forbidden from overtly or covertly saying words; instead, they were asked to say “okay” after each of these words. Then, in each *Restudy* trial, participants were shown a picture on screen as they heard the pseudoword associated with the picture. Once the trial was done, the microphone icon on screen went red, participants had to say “okay” while the icon was still red (3 s). They clicked a button to move to the next trial. For the *Reproduce* condition, participants were asked to overtly say the word they heard. Then, in each *Reproduce* trial, participants were shown a picture on screen as they heard the pseudoword associated with the picture. When the microphone icon turned red, they had to repeat the word. No feedback was provided; participants just clicked a button to move to the next trial. Finally, in the cued *Recall* condition, participants were told to overtly say the name of the picture. Therefore, in each of the cued *Recall* trials, participants were shown a picture on screen (they did not hear anything). The microphone icon went red, and they had 3 seconds to say the word. No feedback was provided; they simply clicked a button to move to the next trial.

Participants learned the names of the pictures in blocks of 9. The first two blocks consisted of exposure to the first category [Pseudoword List 1+ Picture List X], followed by a training block. This structure of two blocks of exposure followed by a block of training was then repeated for the second [Pseudoword List 2 + Picture List Y] and third category [Pseudoword List 3 + Picture List Z]. Each picture category was associated with a different training condition. After the first nine blocks, participants were given a block of exposure and a block of training for each of the three categories, and this sequence was further repeated twice. Thus, there were five passive exposures to each word and four active training trials. In the switch between categories, participants were given an unrelated matching game to complete, where they tried to remember the location of two matching pictures on a grid. This was to avoid strong interference effects between pseudoword lists.

### Cued recall test

Participants completed 27 trials to assess cued recall of the words they had learnt. Trials were blocked by category: within each category, the nine pictures from each category were ordered randomly. In each trial, a picture of the target was shown on the left side of the screen. When ready to respond, the participant pressed the microphone button on the right side of the screen and spoke their response. They were instructed to guess if possible, and say ‘pass’ if they could not recall the word. There was no limit to the time they could take to press the microphone button, but once they pressed this button, they only had 3 s to articulate their response. A further button press then moved on to the next trial.

### Recognition test

Participants also completed 27 trials of a 3-alternative forced choice task. Trials were blocked by category. Each trial showed a picture of a speaker with three buttons underneath. Each button showed a picture drawn from the target set (creatures, plants, or shells). One of the pictures was the target word and the other two were foils. The speaker lit up as the target word was said and participants were asked to choose the matching picture as quickly as they could after they heard the target. The buttons could not be clicked till the sound had stopped playing, to ensure that participants only made their choice once the pseudoword had been said. Items were scored 1 for accurate answers and 0 for inaccurate choices, and this was averaged by category; chance level for this task would be 0.33.

### Testing schedule

Participants completed two sessions spaced exactly 1 week apart, each of which was roughly an hour long. During the first session, they provided demographic details and then completed the word learning game. Immediately after they had finished the training phase, they completed the first cued recall and recognition tests. Recognition was always completed after the cued recall test so participants could not use recent exposure to phonological forms to improve their cued recall performance. Participants were then given a short questionnaire to assess if they had used any strategies to complete the game and if they were familiar with any of the words or pictures in the test. They were then provided with a questionnaire about their language background (LEAP-Q; [47]). If participants had time, they completed this questionnaire by the end of session 1. At the start of the second session, participants were given the cued recall and recognition tests for a second time (cued recall was completed before recognition). Once they completed these sub-tests, they were presented with the first phase of the CVLT-II. They then completed the WASI Matrix Reasoning subtest, the remainder of the LEAP-Q, and were debriefed about the purpose of the learning game. About 30 min after they completed the initial phase of CVLT-II, they completed the late phase and were then paid for their time.

### Data coding and reliability

We scored all audio-recorded productions during the cued recall phase as accurate (1) or inaccurate (0). These were then averaged to calculate cued recall accuracy over the different levels of syllable and training condition. A second rater coded all the words produced in the cued recall condition.

We also calculated normalised Levenshtein distance (normLD) scores between the presented sequence and participant cued recall for each of these words. The Levenshtein distance is the smallest number of edit operations (insertion, substitution, or deletion of a single character) necessary to modify one string to obtain another. By transcribing this data using the International Phonetic Alphabet, we calculated LD in phonemic units. We then normalised this score, using the formula normLD = 1 – LD(P,R)/N, where LD is the Levenshtein distance between P, the presented sequence, and R, the recalled sequence. N is the number of units in the sequence (for further details on normalisation, see [50]). We found that normLD scores were strongly correlated with accuracy scores over all participants, *r* = 0.96, *p* < .0001. As accuracy is a more ecologically valid measure, we avoided conducting further analyses using the normalised Levenshtein’s distance. However, as these might be of future interest, normLD scores are available in the data tables on the Open Science Framework.

## Results

We report results for the recall and recognition tests immediately after training (Week 0) and 7 days after training (Week 1). We present results for training condition and syllable length separately, as we had no hypotheses about an interaction between these two factors. First, the dependent measures of accuracy on cued recall and recognition tests were compared/analysed using repeated measures ANOVAs with time (Week 0/Week 1) and training condition (cued Recall/Reproduce/Restudy) as within-subjects factors. We then analysed the same dependent measures using repeated measures ANOVAs with time (Week 0/Week 1) and syllable length (2/3/4) as within-subjects factors. Generalised effect sizes are calculated using the “afex” package implemented in R version 3.3.0. Any significant main effects or interactions (*p* < 0.05) were followed up with t-tests. All other analyses are reported in exploratory results. We present results from the classical hypothesis testing analyses that we pre-registered and if significant would allow us to reject the null hypothesis, and then report additional Bayesian analyses (using JASP 0.7.5.6; JASP Team, 2016) so we can compare the weight of evidence in support of the null hypothesis with that in support of the alternative.

### Results for training condition (fig. 2)

*Cued Recall*: Taken together, our first two hypotheses were that we would observe a time x condition interaction on the accuracy measure of cued recall testing. We conducted a repeated-measures ANOVA with time (Week 0/ Week 1) and condition (*Recall*/*Reproduce*/*Restudy*) as factors. We found a significant main effect of time, *F* (1,35) = 45.32, *p* < .001, *η* ^*2*^_*G*_ = .09, with participants showing evidence of forgetting (i.e. they were less accurate) between Week 0 (M = 0.53, SD = 0.24) and Week 1 (M = 0.36, SD = 0.22). Both the main effect of condition, *F*(2,70) = 2.68, *p* = .076, *η* ^*2*^_*G*_ = .01 and the interaction between time and condition, *F*(2,70) = 1.37, *p* = .261, *η* ^*2*^_*G*_ = .002 were not significant. The data were also examined by using Bayesian analyses [51], which allows us to avoid some statistical issues related to *p*-values (for example, the setting an arbitrary criterion to achieve significance [52]). The Bayesian approach adopted employs Bayes factors to compare support for the alternative or experimental hypothesis relative to the null hypothesis. We use the default priors implemented in JASP v 0.7.5.6 [53] for a Bayesian repeated-measures ANOVA [54], and estimate the Bayes inclusion factor. This is the Bayes factor averaged across all the models that include the effect of interest, compared to all the models that do not include this effect. The estimated Bayes inclusion factor (BF_10_) for the effect of time indicated that the data were approximately 4 × 10^8^:1 times in favour of the alternative hypothesis (relative to all alternative models). This would be considered decisive evidence for the alternative hypothesis [55]. The Bayesian inclusion factors (BF_10_) for condition and the interaction between time and condition were 1.059 and 0.343 respectively, suggesting that the evidence for the inclusion of condition or the interaction between time and condition in the model was inconclusive (note that this does not indicate support for the null hypothesis).

**Fig. 2:**
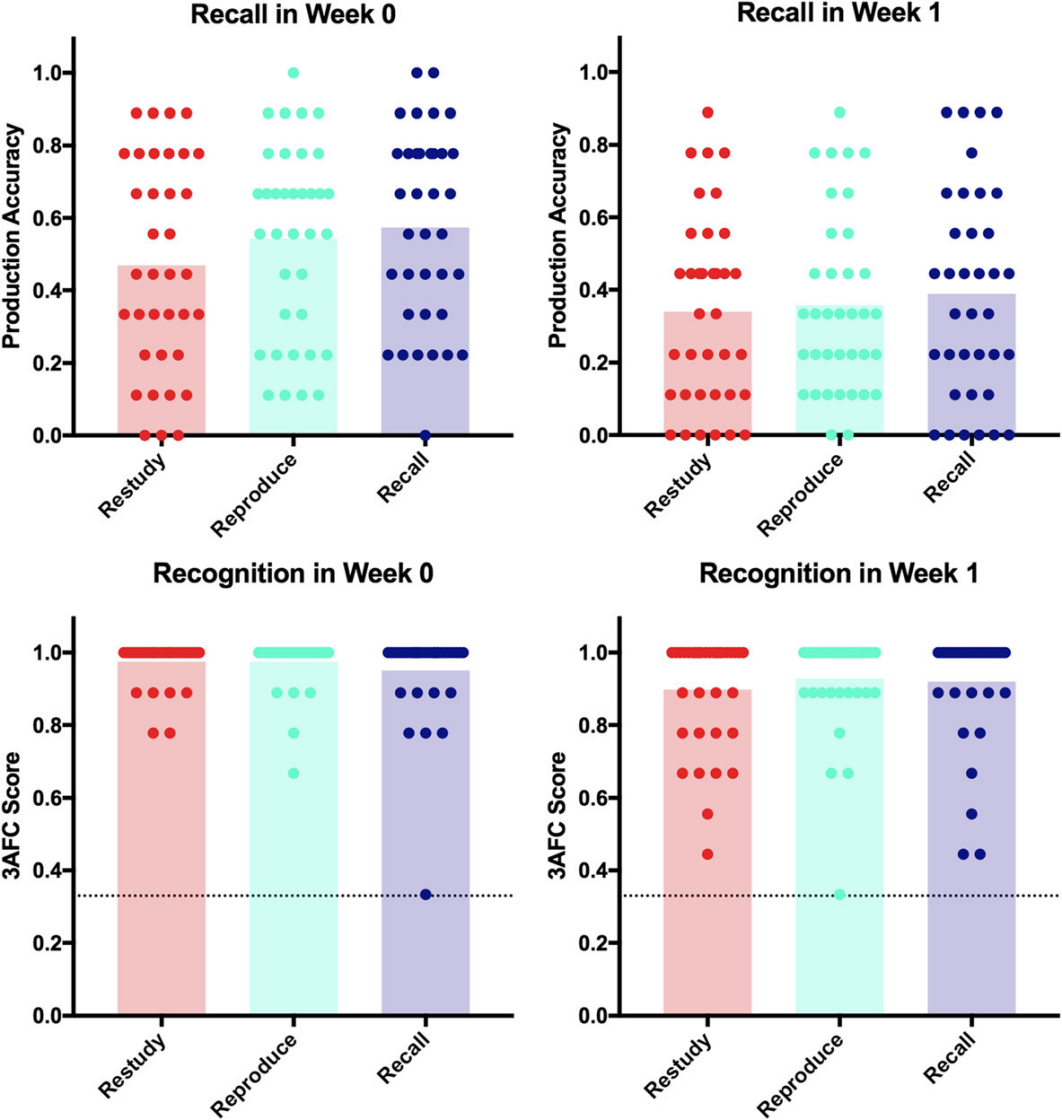
Effect of Training Condition on recall and recognition. The bars show the mean accuracy in each training condition over Week 0 and Week 1. Individual datapoints show the score achieved by each participant by condition. The line at the top of the bars in the recognition graphs represents data from participants who were 100% accurate; which are not individually identifiable due to the large number. The dotted line on the recognition graphs denotes chance

#### Recognition

We note that recognition accuracy scores showed ceiling effects, with mean scores in all categories being >/=90%. We therefore did not run parametric statistics on these measures.

### Results for word length (fig. 3)

#### Recall

To test hypothesis 3, which posits that accuracy will be greater for shorter pseudowords (less complex) relative to longer pseudowords (more complex), we conducted a repeated-measures ANOVA with Time (Week 0/ Week 1) and Word length (2/3/4) as factors. We found a significant effect of time, *F* (1,35) = 45.32, *p* < .001, *η* ^*2*^_*G*_ = .09, word length, *F* (2,70) = 38.23, *p* < .001, *η* ^*2*^_*G*_ = .15, and an interaction between time and word length, *F* (2,70) = 9.43, *p* < .001, *η* ^*2*^_*G*_ = .02. The main effect of time is as noted above. For word length, participants were more accurate at producing shorter words, with scores for 2-syllable (M = 0.58, SD = 0.25), 3-syllable (M = 0.44, SD = 0.27), and 4-syllable words, (M = 0.31, SD = 0.21), all being significantly different from each other (*p* < .001 for all comparisons). The interaction was driven by a reduced rate of forgetting between Week 0 and Week 1 for the 4-syllable words, relative to the 2-syllable and 3-syllable words. The Bayesian inclusion factors indicated decisive to strong effects for all three factors, Time (7.4 × 10^9^), Syllable Length (7.51 × 10^14^) and Time x Syllable (20.96).

**Fig. 3:**
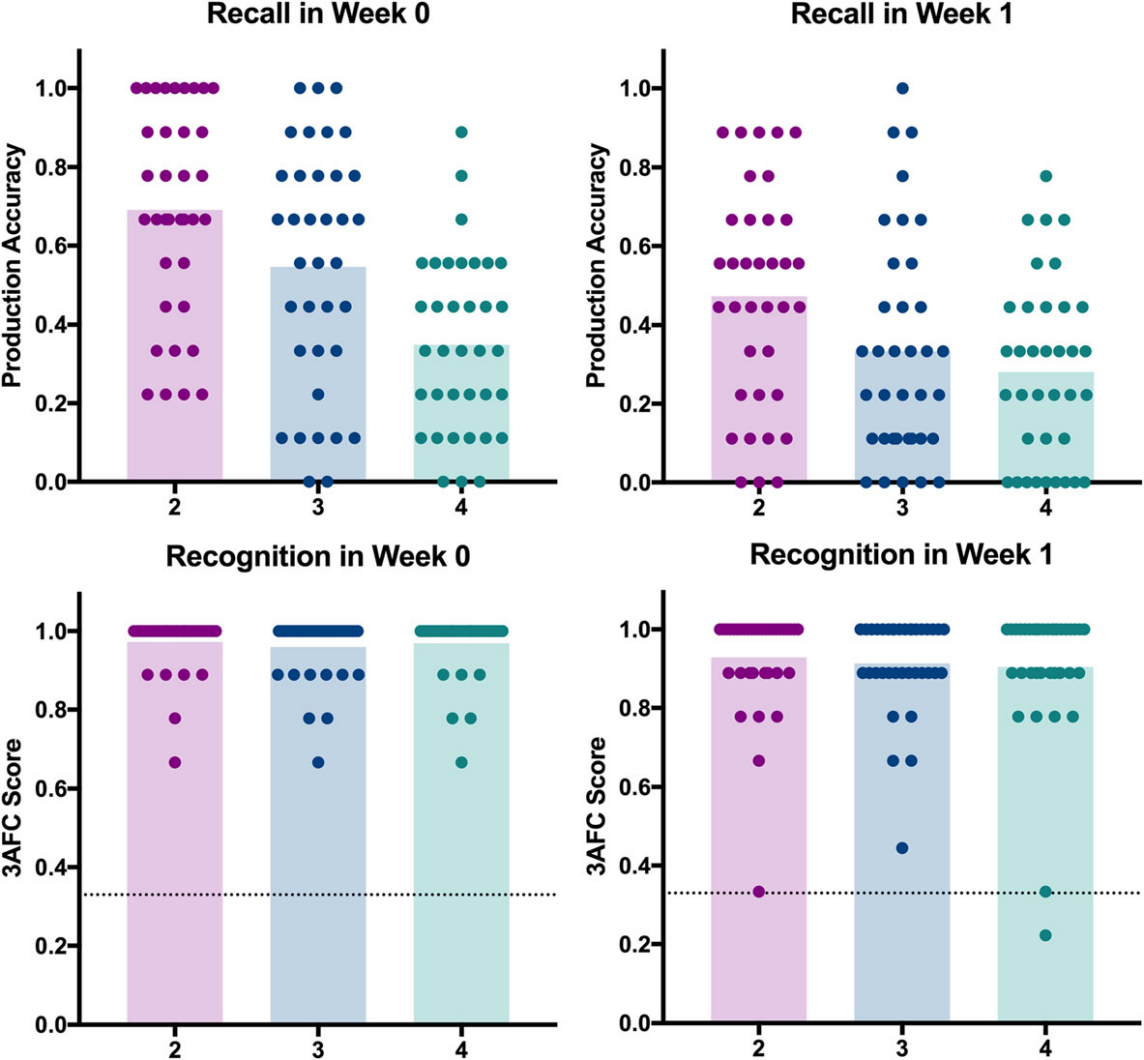
Effect of Word Length on recall and recognition. The bars show the average accuracy for each syllable length over Week 0 and Week 1. Individual datapoints show the score achieved by each participant by condition. The line at the top of the bars in the recognition graphs represents data from participants who were 100% accurate; which are not individually identifiable due to the large number. The dotted line on the recognition graphs denotes chance

#### Recognition

Again, we note that recognition accuracy scores showed ceiling effects, with average scores in all categories being >90%. Therefore, we did not use parametric analysis of variance to analyse this data.

### Exploratory analyses

#### Evaluating evidence for hypotheses 1 and 2

In our pre-registered analysis, we only planned to conduct follow-up t-tests if the main effects of training condition or the interaction between training condition and time were significant. This was not the case. However, in these exploratory analyses, we sought to quantify specific evidence for hypotheses 1 and 2 using t-tests, and assess in what ways the data looked different from our predictions.

Hypothesis 1 posited that training conditions with more listening exposures to stimuli (*Reproduce* and *Restudy*) would lead to greater accuracy immediately after training relative to the condition where they received fewer exposures (cued *Recall)*. To evaluate this hypothesis, separate paired t-tests were used to compare the accuracy scores for cued recall test at week 0 of stimuli studied under the condition of cued Recall with those studied under the Restudy and the Reproduce conditions. We found significantly higher accuracy at recall test for words studied in the *Recall* relative to the *Restudy* condition, *t* (35) = 2.86, *p* = .007. The estimated Bayes factor (null/alternative) indicated that the data were more than 5 times more likely to occur under the model that included a difference among training conditions, rather than the model that did not include this factor. However, it is important to note that this difference was in the opposite direction to that predicted, in that participants were less accurate in the condition where they had to *Restudy* words (M = 0.47, SD = 0.29), relative to the cued *Recall* condition (M = 0.57, SD = 0.26), which had the fewest exposures. There was no significant difference in accuracy for stimuli learnt in the *Recall* condition compared with the *Reproduce* condition (M = 0.54, SD = 0.26), *t* (35) = 1.01, *p* = .319. The estimated Bayes factor indicated that the data were approximately 3.5:1 in favour of the null hypothesis for this contrast.

Hypothesis 2 stated that accuracy at Week 1 would be highest for the words studied under the Recall condition relative to the other two conditions. We tested this using separate t-tests with cued recall accuracy as a dependent measure. There was no significant difference between the cued Recall and *Restudy* conditions, *t* (35) = 1.24, *p* = .222, or the *Recall* and *Reproduce* conditions, *t* (35) = 0.83, *p* = .412. The estimated Bayes factors indicated that these data were approximately 2.7:1 and 4:1 (respectively), or weak evidence in favour of the null hypotheses.

#### Replication of production effect

We considered whether we could replicate the classic production effect reported for written words, that is, a benefit of learning words in the *Reproduce* relative to the *Restudy* condition. This effect is traditionally observed in tests immediately following the production trials. Using a one-tailed paired t-test, we examined whether the *Reproduction* condition resulted in higher accuracy for cued Recall test scores than the *Restudy* condition at Week 0. We found a one-tailed statistically significant difference between these two measures, *t* (35) = 1.81, *p* = .039. Using a directional Bayesian paired t-test (testing if accuracy in the *Reproduction* condition exceeded accuracy in the *Restudy* condition, excluding effects in the opposite direction), the Bayes Factor was estimated to be 0.784:1 in favour of the alternative hypothesis, or 1.5 times more likely to occur under the production effect than a chance occurrence. This constitutes only inconclusive evidence in support of the alternative hypothesis. At week 1, a one-tailed t-test comparing performance in the *Reproduction* and *Restudy* conditions was not significant, *t* (35) = .40, *p* = .345. Using a one-tailed Bayesian paired t-test, the Bayes Factor was estimated to be 0.251:1 in favour of the alternative hypothesis, or 3.98 times more likely to be a chance occurrence than the production effect. This is weak evidence in support of the null.

#### Correlations between ability and performance

We also examined possible relationships between IQ, verbal memory, age and word learning ability (as assessed in Week 0). We found pairwise correlations between word learning ability and age, *r(35)* = −0.39, *p* = .019, indicating that getting older was associated with poorer word learning performance, although note that our age range (20-39 years) was quite limited; between learning ability and nonverbal reasoning ability, *r(35)* = .34, *p* = .046, where higher nonverbal IQs were associated with better word learning (again, this was a relatively restricted range, with standard scores ranging between 47 and 68); and between free recall scores on the CVLT-II and word learning, *r(35)* = .53, *p* < .001, indicating that participants with better verbal recall showed better pseudoword learning. These correlations all remain significant (*p* < 0.05) when we apply a Holm correction for 3 comparisons. A model including all of these factors was significant, adj-R^2^ = .32, *p* = .0014 with verbal memory scores (*p* = .023) accounting for unique variance in the model (change in adj-R^2^ = .09). Age and IQ did not account for significant variance in this model.

## Discussion

Overall, recognition accuracy was high immediately after training and on the delayed test that followed a week after training, indicating that participants were able to form associations between the novel picture sets and pseudowords. As participants were at ceiling on recognition, the recognition task was not useful for exploring differences between training conditions, and we discuss the remainder of the results only with respect to cued recall performance (assessed by oral production of the target pseudoword in response to the visual referent). We had predicted that accuracy in the cued *Recall* condition would be worse than in the *Reproduce* and *Restudy* conditions immediately after training, but the pattern of data for cued recall accuracy went opposite to prediction, with an advantage for the cued *Recall* over the *Restudy* condition (though not over the *Reproduce* condition). In addition, contrary to our prediction, there was no boost for the cued *Recall* condition over time relative to the other two conditions. Rather, we found that participants forgot all words over time regardless of the training condition. Thus active and effortful manipulation of words, both via retrieval and reproduction, relative to passively listening to words, did not confer a long-term learning advantage.

We treated the word-length effect as a positive control, i.e. to establish that our experimental methods yielded typical effects. In this case the prediction was that shorter words should be easier to learn than longer words. There was strong evidence of this effect in the production task, indicating that we were both adequately powered and measuring relevant aspects of phonological and speech motor learning. While previous work has shown that the word-length effect is a stable and robust phenomenon [44, 56], we also show that this effect persists over a period of 1 week. These findings are unsurprising – the longest words were associated with the lowest production accuracy at both immediate testing and after a delay of 1 week. We did observe a syllable length x time condition that we did not predict. This appears to suggest that once a longer 4-syllable item is encoded, it is more resistant to forgetting. Further testing is required to confirm whether this effect is specific to the words we included, or whether this would generalise to other samples.

In contrast to word length, we found mixed evidence for testing and production effects. Although there was only inconclusive evidence in support of a time x condition interaction, exploratory testing for our specific hypotheses allows us to shed some light on the pattern of data we observed when testing immediately after training, and a week following training. We found some support for the testing effect and the production effect immediately after training (these are discussed below). Yet, despite this initial pattern of results, there was no evidence to suggest that testing or production benefits persisted a week after training. First, the testing enhancement did not translate into better retention at the one-week re-test. This means that we did not replicate the classic testing effect, which is associated with not just better performance but reduced forgetting [6, 11, 16]. Second, unlike Ozubko and colleagues [34], we observed no beneficial effect of production over a longer time-span. The lack of these differences might be accounted for by procedural variations between previous training tasks and the one we employed. For instance, the lack of a sustained benefit of cued *Recall* over *Restudy* at Week 1 might be because we tested memory at a different stage of encoding. In the Karpicke and Roediger [6] study, participants were allowed to learn until they achieved correct recall of the target-response pairing, and only at this stage were recall or restudy regimes put in place. In contrast, we used the same number of exposure trials for all conditions, and this may have led to a reduced performance boost for the test condition. The long-term production advantage in the Ozbuko et al. [34] study emerged when examining recognition of known words, not recall of novel pseudowords. Another explanation for the pattern of results we observed one-week following training is that the difference between the conditions was not ‘pure’, as providing recall on a set of words may have encouraged participants to covertly use this strategy on all words, despite the instructions we provided. However, the fact that we did observe a difference between conditions immediately after training would temper this argument. Another possible influence on our results at Week 1 is the fact that we tested all words immediately after training. Single instances of testing have been shown in previous studies to lead to an improvement in performance [57, 58]. It is possible that we enhanced learning in the *Restudy* and *Reproduce* conditions by providing an opportunity to practice retrieval of these words by testing performance on all words immediately after training. The bifurcation hypothesis [25] predicts that successful retrieval of items at the final test immediately after training would confer a substantial advantage to these items. Therefore, by assessing performance of words learned in the non-retrieval conditions, we may have inadvertently provided a retrieval opportunity, which provided a learning boost in these conditions. This may have reduced the difference between effects in the ‘Recall’ and ‘Reproduce’/‘Restudy’ conditions at Week 1. In order to explore this question more carefully, it would be necessary to use a design where only half of the words from all conditions were tested immediately after training, and then all the words were tested a week after training. If the single instance of retrieval is of benefit, then the untested words in the *Reproduce* and *Restudy* conditions would show no enhancement. However, it is worth highlighting that this would suggest that a single instance of cued *Recall* had the same effect as five instances of cued *Recall* with interleaved exposure, which is somewhat unlikely.

A goal of this study was to assess whether tasks purported to rely on different neurobiological pathways lead to differences in behavioural accuracy, but we did not find any such differences in healthy adults. We note that the lack of behavioural differences does not argue against the use of different neurobiological pathways to accomplish this learning. In healthy adults, it is entirely possible that learning via different pathways offers the same learning benefits. Therefore, the ideal way to address this question would be to use similar tasks with populations where one of the learning pathways is compromised. In the learning literature, this is typically done by the examining performance of participants with Parkinson’s disease (which results from depletion of dopaminergic input to the striatum) or participants with medial temporal lobe damage. Thus, this is an issue to be addressed in future studies, using either patient groups, or using drug manipulations that affect the functioning of dopaminergic systems.

### Discussion of findings from exploratory analyses

#### Testing effect immediately after training

We did observe a strong testing effect immediately after training, although we note that the direction of the effect was contrary to our predictions [16]. However, our result is consistent with the bifurcation hypothesis [25], which maintains that successfully retrieved items receive a boost that restudied items do not, so that high exposure to items might allow a testing enhancement to be observed at short intervals. Given the nature of our testing materials, this enhancement is unlikely to result from elaborative semantic processing, as both the visual referent and the phonological form were unfamiliar to the participant. More suitable explanations are offered by retrieval effort hypotheses [22] discussed earlier.

#### Production effect immediately after training

We found weak or anecdotal evidence in favour of the production effect *(Reproduce > Restudy*) immediately after learning [15]. Procedural variations introduced in our study may have influenced the strength of the production effect. For example, participants in this study produced overt speech in the contrasting conditions, (“okay” for *Restudy*, and the full word for *Recall*), rather than staying silent. This may have led to better performance by improving attention to all words. Alternatively, participants may have covertly applied reproduction or recall strategies that were required in other blocks. There is some evidence that covert retrieval is as effective as overt retrieval [14], and that covert reproduction involves the same mechanisms as overt reproduction [40]. However, it is somewhat unlikely that participants applied a retrieval strategy to the ‘*Restudy’* condition. Participants typically do not assume that testing leads to more effective learning [6], and therefore they would be unlikely to apply this strategy more broadly. In addition, our rationale for having the participants say “okay” was to limit covert reproduction. Finally, we found that performance on stimuli learnt under the cued *Recall* and *Reproduce* conditions was indistinguishable when tested immediately after training. This result was not what we hypothesised and contrasts with previous findings in foreign vocabulary learning in which practice with recall results in greater learning accuracy [8]. This suggests that the similarities between the cued *Recall* and *Reproduce* conditions (encoding and producing the word form) may have been more important than their differences (retrieval mode, level of cognitive processing). Conversely, saying “okay” in the *Restudy* condition may have acted as a form of articulatory suppression [59], which reduced performance for stimuli learnt in this condition.

#### Individual differences in cued recall accuracy

A factor that further distinguishes our study from previous work on the testing and production effects is the focus on oral production rather than testing recall via written means [6, 9]. Despite testing healthy young adults, we found a great deal of individual variation with respect to production performance. We found that some participants were unable to recall accurately any of the words they had just learned, despite receiving at least five auditory exposures. On the other hand, some participants could accurately recall all of the words. Despite the individual variation in production, most participants performed at >90% when it came to recognition. This indicates that participants were able to match words to their referents. Consequently, the individual variation in production must stem from the phonological and motor aspects of novel word learning. This is a non-trivial process even in adulthood, especially when a phonological form has to be learned aurally, and cannot be derived from existing vocabulary. About 32% of the variation in learning was explained by verbal memory. The fact that the verbal recall of a list of words predicted performance on novel word learning suggests that short term memory and chunking processes may aid learning. So how could such phonological learning be improved? Previous developmental studies have also suggested that learners benefit from the presence of orthography [60], which may help learners segment phonological chunks in novel words. Future studies that assess whether participants are better able to learn these words when they are presented in their written form, and if recall accuracy differs across the spoken and written form, are warranted. Other studies have also suggested that feedback can enhance the testing effect [61, 62], although many of these studies do not test the learning of novel phonology. In our paradigm, even though participants had a chance to receive further exposures to a target-referent pair, no corrective feedback was given. It is possible that providing feedback on participants’ oral production could improve their phonological learning, and consequently their cued recall of the phonological targets.

## Conclusions

In summary, it is clear that our primary hypothesis about training conditions conferring specific advantages for oral vocabulary learning was not supported by our data. In other words, the results from our study suggest that in training expressive vocabulary, reproducing, recalling or restudying a word leads to similar production accuracy over the long term. There may, of course, be practical reasons to prefer one training method over another: a busy teacher might find that it is far easier to get students to imitate new words rather than designing tests for recall practice. Students might prefer restudying to the anxiety associated with tests.

We used one specific training paradigm and it is possible that variations in procedure could lead to one of these conditions inducing better learning. For example, providing only one of set of instructions to a participant and assessing between-subject effects to maintain purity of condition, or providing feedback on performance [62] might serve to enhance the effects these conditions. Changing the set size or the phonotactics of the words to be learned might also lead to a different pattern of results. Nevertheless, our findings indicate that we cannot assume that classic training effects will generalise beyond the written paradigms that are typically used. This is particularly important for translational purposes. A recent study found that memory strategies that were robust in laboratory settings could not be replicated in real-world settings such as classrooms [63]. The authors argued this could be because of increased noise, the presence of other tasks, and the overall performance difficulty of conditions associated with better learning in the lab. Therefore, pinning down both what does and does not work is equally important to help us assess what training conditions could confer benefit in clinical and educational settings.

## Footnotes

1 In the memory literature, recognition is the process involving detection of familiarity with an event or item, whereas recall involves the retrieval of related details from memory.

2 The term retrieval can refer to both recognition and recall.
